# rTMS of the Left Dorsolateral Prefrontal Cortex Modulates Dopamine Release in the Ipsilateral Anterior Cingulate Cortex and Orbitofrontal Cortex

**DOI:** 10.1371/journal.pone.0006725

**Published:** 2009-08-21

**Authors:** Sang Soo Cho, Antonio P. Strafella

**Affiliations:** 1 Toronto Western Research Institute and Hospital, UHN, University of Toronto, Toronto, Canada; 2 PET Imaging Centre, Centre for Addiction and Mental Health, University of Toronto, Toronto, Canada; The University of Western Ontario, Canada

## Abstract

**Background:**

Brain dopamine is implicated in the regulation of movement, attention, reward and learning and plays an important role in Parkinson's disease, schizophrenia and drug addiction. Animal experiments have demonstrated that brain stimulation is able to induce significant dopaminergic changes in extrastriatal areas. Given the up-growing interest of non-invasive brain stimulation as potential tool for treatment of neurological and psychiatric disorders, it would be critical to investigate dopaminergic functional interactions in the prefrontal cortex and more in particular the effect of dorsolateral prefrontal cortex (DLPFC) (areas 9/46) stimulation on prefrontal dopamine (DA).

**Methodology/Principal Findings:**

Healthy volunteers were studied with a high-affinity DA D2-receptor radioligand, [^11^C]FLB 457-PET following 10 Hz repetitive transcranial magnetic stimulation (rTMS) of the left and right DLPFC. rTMS on the left DLPFC induced a significant reduction in [^11^C]FLB 457 binding potential (BP) in the ipsilateral subgenual anterior cingulate cortex (ACC) (BA 25/12), pregenual ACC (BA 32) and medial orbitofrontal cortex (BA 11). There were no significant changes in [^11^C]FLB 457 BP following right DLPFC rTMS.

**Conclusions/Significance:**

To our knowledge, this is the first study to provide evidence of extrastriatal DA modulation following acute rTMS of DLPFC with its effect limited to the specific areas of medial prefrontal cortex. [^11^C]FLB 457-PET combined with rTMS may allow to explore the neurochemical functions of specific cortical neural networks and help to identify the neurobiological effects of TMS for the treatment of different neurological and psychiatric diseases.

## Introduction

Brain dopamine (DA) is implicated in the regulation of movement, attention, reward and learning [1]–[3] and plays an important role in Parkinson's disease, schizophrenia and drug addiction [4]–[5]. Several animal and human studies have demonstrated that the frontal cortex exerts an important influence on striatal dopamine release both through the modulation of dopaminergic neuronal firing [6]–[7] and a direct effect on nerve terminals [8]–[14]. While large evidence exists on the control exerted by the frontal cortex on striatal dopamine, very little is known about the modulation of dopamine within the prefrontal cortex. Animal experiments have demonstrated that brain stimulation is able to induce significant dopaminergic changes in extrastriatal cortical areas [10], [15]–[18]. So far, not many studies have been able to address this issue directly in the human brain.

Given the up-growing interest of non-invasive brain stimulation as potential tool for treatment of neurological and psychiatric disorders [19]–[21], it would be critical to investigate dopaminergic functional interactions in the prefrontal cortex and in particular the effect of dorsolateral prefrontal cortex (DLPFC) (areas 9/46) stimulation on prefrontal dopamine. The DLPFC is heavily interconnected with several cortical and subcortical areas [22]–[24]. In particular, anatomical and functional connections between DLPFC and medial prefrontal cortex, including anterior cingulate cortex (ACC) and orbitofrontal cortex, have been documented by anatomical and neuroimaging studies [25]–[29].

Today, the possibility of modulating the activation of a given cortical area in the human brain by using repetitive transcranial magnetic stimulation (rTMS) combined with positron emission tomography (PET) provides us with a valuable probe of brain function to investigate neural loops in human subjects. So far, commonly used PET radioligands (i.e. [^11^C]raclopride) for examination of DA-D2 receptors have not been useful in providing sufficient signal-to-noise ratio in regions outside the striatum with low D2-dopamine receptor density [30]. The development of novel high-affinity ligands such as [^11^C]FLB 457 (K_D_ = 20pM) has overcome this limitation and is enabling us to investigate the extrastriatal dopaminergic system. These dopaminergic ligands have shown significant dopaminergic changes in several cortical areas during both pharmacological challenge [31]–[34] and cognitive tasks [35]–[37].

In this study, we used PET ligand [^11^C]FLB 457 to map and quantify the effect of left and right DLPFC rTMS on the ipsilateral prefrontal dopamine with the specific objectives of identifying 1) dopaminergic changes in the ipsilateral medial prefrontal cortex (i.e. ACC and orbitofrontal cortex) and 2) potential differences and/or asymmetries between the two hemispheres.

## Results

**Figure 1:**
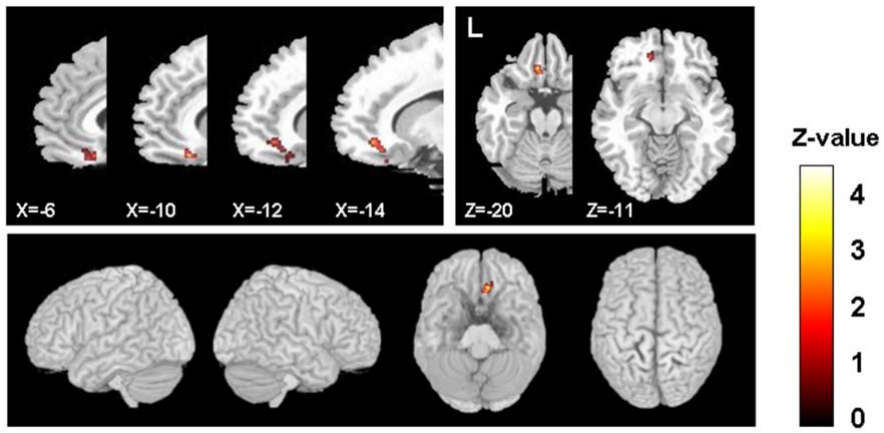
Sagittal and transverse sections of the statistical parametric map of the changes in [^11^C]FLB 457 BP after left DLPFC stimulation overlaid upon the standard MRI.

Parametric image analysis revealed significant differences between left and right hemisphere. Table 1 summarizes the main findings. rTMS on the left DLPFC induced a specific reduction in [^11^C]FLB 457 binding potential (BP) in the ipsilateral subgenual ACC (BA 25/12), pregenual ACC (BA 32) and medial orbitofrontal gyrus (BA 11), likely reflecting dopamine release in those areas (Figure 1). There was no statistically significant BP difference in any other cortical areas. The same stimulation paradigm applied over the right DLPFC did not induce any detectable changes in [^11^C]FLB 457 BP in the medial prefrontal cortex or other cortical regions.

**Table 1:** Prefrontal regions with reduction in [^11^C]FLB 457 BP following left DLPFC stimulation.

Region	BA	coordinates* x, y, z	z-score	SVC corrected-*P* value	Cluster size (mm^3^)
L subgenual ACC	BA 25/12	−10, 28, −18	4.40	0.003	1432
L medial OFC	BA 11	−6, 30, −20	3.89	0.025	424
L pregenual ACC	BA 32	−14, 40, −10	4.15	0.008	1024

L: left, ACC: anterior cingulated cortex, OFC: orbitofrontal cortex, BA: Brodmann's area *Talairach coordinate (mm), SVC: small volume correction.

**Figure 2:**
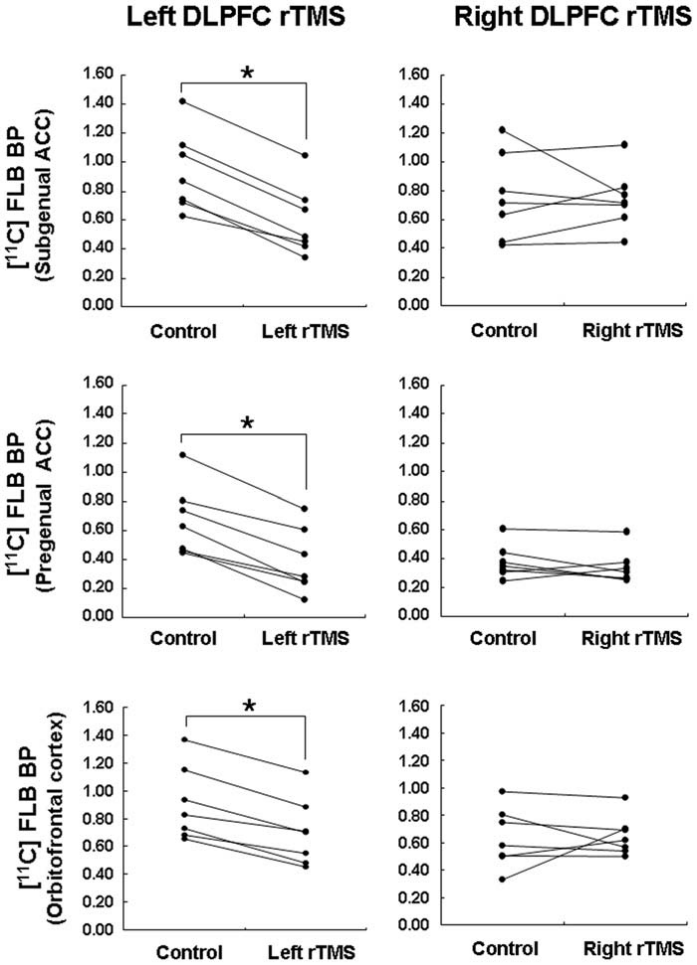
Individual mean BPs extracted from 10 mm radius VOI. Upper, middle and lower figures show the individual [^11^C]FLB 457 BP changes the subgenual ACC (x = −10, y = 28, z = −18), pregenual ACC (x = −14, y = 40, z = −10) and medial orbitofrontal cortex (x = −6, y = 30, z = −20) under control and active rTMS conditions, respectively.

Individual BPs extracted from ROI centered at the statistical peak defined by the parametric maps (subgenual ACC: x = −10, y = 28, z = −18; pregenual ACC: x = −14, y = 40, z = −10; medial orbitofrontal cortex: x = −6, y = 30, z = −20) confirmed these findings and showed significant BP reduction following left but not right DLPFC rTMS (Table 2 and Figure 2). Repeated-measures ANOVA revealed a significant interaction between rTMS site (left−right DLPFC)×ROI site (left−right ROI) in the subgenual ACC (*F*(1, 6) = 15.01, *P* = 0.008), the pregenual ACC (*F*(1, 6) = 155.64, *P* = 0.00002) and orbitofrontal gyrus (*F*(1, 6) = 6.64, *P = *0.04).

**Table 2:** Mean [^11^C]FLB 457 BP changes in prefrontal regions following left and right DLPFC.

	Left DLPFC rTMS	control	*P*	Right DLPFC rTMS	control	*P*
subgenual ACC	0.93 (0.28)	0.59 (0.24)	<0.001*	0.74 (0.21)	0.76 (0.30)	0.9
pregenual ACC	0.67 (0.24)	0.38 (0.22)	<0.001*	0.34 (0.12)	0.38 (0.12)	0.3
medial OFC	0.90 (0.27)	0.69 (0.24)	<0.001*	0.63 (0.22)	0.65 (0.15)	0.9

ACC: anterior cingulated cortex, OFC: orbitofrontal cortex, DLPFC: dorsolateral prefrontal cortex, Number in brackets indicates S.D.

*Bonferroni correction.

The post-hoc paired t-test confirmed a significant reduction in [^11^C]FLB 457 BP compare to control condition following left DLPFC rTMS in the ipsilateral the subgenual ACC (mean±SD: 0.93±0.28 vs 0.59±0.24; t = 11.2, *P*<0.001), pregenual ACC (mean±SD: 0.67±0.24 vs 0.38±0.22; t = 8.4, *P*<0.001) and medial orbitofrontal cortex (mean±SD: 0.90±0.27 vs 0.69±0.24; t = 9.1, *P*<0.001). The mean magnitude of decrease in [^11^C]FLB 457 BP was −37.7±9.7% for subgenual ACC, −45.3±17.1% for pregenual ACC, and −23.6±7.4% for medial orbitofrontal cortex. In contrast, no significant BP changes were seen following stimulation of the right DLPFC compared to the control condition in the right subgenual ACC (x = 10, y = 28, z = −18, 0.74±0.21 vs 0.76±0.30, t = 0.2, *N.S*), right the pregenual ACC (x = 14, y = 40, z = −10, 0.34±0.12 vs 0.38±0.12, t = 1.1, *N.S*), and right medial orbitofrontal cortex (x = 6, y = 30, z = −20, 0.63±0.22 vs 0.65±0.15, t = 0.20, *N.S*) (Table 2 and Figure 2).

To rule out a potential behavioral contribution to these findings, behavioral ratings before and after left or right DLPFC rTMS were measured and results are presented in Table 3. Statistical analysis of behavioral measures revealed no significant main effect of the stimulation site (left or right DLPFC rTMS) and rTMS condition (before and after rTMS) and interaction in all behavioral aspect.

**Table 3:** Mean behavioral ratings before and after rTMS of the left and right DLPFC.

	Pre Left DLPFC rTMS	Post Left DLPFC rTMS	*P*	Pre Right DLPFC rTMS	Post Right DLPFC rTMS	*P*
Discomfort-comfort	1.29 (1.89)	0.43 (1.90)	N.S	1.57 (0.79)	0.43 (1.51)	N.S
Fatigued-rested	−0.14 (1.57)	−0.29 (1.80)	N.S	−0.43 (1.27)	0.71 (1.11)	N.S
Anxious-calm	−0.14 (1.86)	0.14 (2.04)	N.S	0.43 (1.13)	0.43 (1.62)	N.S
Sad-happy	1.29 (1.11)	1.43 (0.79)	N.S	1.14 (1.46)	1.43 (1.13)	N.S
Irritated-soothed	−0.43 (1.90)	0.57 (1.27)	N.S	−0.29 (1.89)	0.29 (1.38)	N.S
Feel pain-do not feel pain	−0.14 (2.27)	0.57 (1.13)	N.S	−0.29 (2.06)	−0.43 (0.98)	N.S

DLPFC: dorsolateral prefrontal cortex, Number in brackets indicates S.D.

## Discussion

The present rTMS/[^11^C]FLB 457 PET study revealed two main findings. First, that DLPFC-rTMS led to focal DA changes in the ipsilateral ACC (subgenual part, BA 25/12 and pregenual part, BA 32) and medial orbitofrontal cortex (BA 11) and second, that while stimulation paradigm and target cortical area were similar among all subjects, only left DLPFC but nor right DLPFC induced significant changes in [^11^C]FLB 457 BP in those areas.

Previous PET studies, have shown that [^11^C]FLB 457 BP may provide a reasonable estimate of receptor densities in different extrastriatal areas including cingulate cortex [38] and may be sensitive in detecting changes in extrastriatal endogenous dopamine concentration [31], [34], [35], [39], [40]. PET animal experiments in baboon monkeys with [^18^F]fallypride (a similar high-affinity dopaminergic radioligand) have confirmed these findings and showed that amphetamine challenge may induce a striking reduction in BP in the ACC (41%) and other extrastriatal areas [41]. Thus, there is large evidence that [^11^C]FLB 457 ligand may be well-suited to capture differences in dopaminergic binding in prefrontal areas.

Recently, the medial prefrontal areas including ACC and orbitofrontal cortex have received particular attention in cognitive and behavioral brain imaging studies. The ACC in particular has emerged as a locus of information processing and regulation [42]. In terms of cytoarchitecture, the ACC is a heterogeneous area in its functions and connectivity [27], [43]. It is involved in motor control, cognition, pain and emotion, arousal and drive [44]. In humans, this brain structure can be divided into caudal and rostral along the vertical plain (Y = 10) and supracallosal and subcallosal runs along the horizontal plain (Z = 2). Particularly, the subcallosal part of the ACC (including pre and subgenual ACC) is linked with DLPFC and orbitofrontal cortex as well as striatal area [27], [42]. This is associated with non-goal directed, stimulus independent processes included within the default mode network comprising the ventromedial prefrontal, posterior cingulate and lateral parietal cortex [45], [46].

The ACC is one of the main target areas of the mesocortical dopamine system originating in the ventral tegmental area (VTA) [1], [47]. The prefrontal cortex sends glutamate containing projection to the VTA in the midbrain influencing the mesocortical ascending dopaminergic pathway [48]–[50]. In particular, Taber and his co-worker found that the glutamatergic projection from prefrontal cortex regulates the activation of dopaminergic neuron in VTA [50]. Thus, it is possible that stimulation of the left prefrontal cortex with rTMS and subsequent activation of VTA via glutamatergic projection can possibly influence VTA dopaminergic neurons and cause dopaminergic changes in ACC/medial orbitofrontal area.

However, it cannot be ruled out a more direct rTMS-induced effect of DLPFC on ACC and orbitofrontal cortex. In fact, because of the dense anatomical connections between DLPFC and ACC [25]–[27], we cannot exclude the possibility that these effects might be mediated by local activation of the nerve terminals. It is interesting to note on this purpose one study done in treatment-resistant depressed patient which demonstrated specific functional interaction between DLPFC and subgenual ACC where deep brain stimulators (DBS) were implanted. In these patients, Mayberg and colleagues showed that DBS of subgenual ACC (in the same location where also our findings were observed) was able to restore activation of the DLPFC showing evidence of a strong functional interaction between these two prefrontal areas [29].

A striking observation of our study was the asymmetric effect on dopamine release between left and right hemisphere. It is known that the rCBF changes induced by prefrontal rTMS differ upon hemisphere stimulated and vary with stimulation frequency [51]. Hemispheric differences of the frontal lobe in anatomy and neurochemistry as well as in cognition and behavior are well described and not unknown in the literature [52]. In fact, differences in laterality involving DLPFC has been shown in several cognitive domains such as inhibition [53], time perception [54] and risk taking behavior [55]. In a recent rTMS/[^11^C]raclopride PET during set-shifting task, our group found a significant hemispheric asymmetry [56]. rTMS of the left DLPFC impaired executive task performance and dopamine release in the ipsilateral striatum while no effects were seen following right DLPFC stimulation. Differences in left and right hemispheric involvement of TMS treatment in patients with depression have also been reported [57], [58]. In a recent review on the efficacy of rTMS in major depression, the large majority of those studies reported significant remission of depression following high-frequency rTMS applied to the left DLPFC [59] while some changes with right DLPFC were seen only when different and lower stimulation frequencies (i.e. 1 Hz) were used. These observations were re-confirmed in another large double-blinded, multisite study, with major depression patients who were randomized to high-frequency rTMS (at 10 Hz) or sham TMS over the left DLPFC and found that active rTMS was effective in treating major depression [60]. Possibly, different neuroanatomical and neurochemical modulations are involved in the left and right prefrontal neural networks to explain such differences in cognition and behavior. On this note, it would be worth to report previous studies showing that left and right 6-hydroxydopamine lesions of the medial prefrontal cortex differentially alter subcortical dopamine utilization and the behavioral response to stress [61]. These important experiments revealed that an intrinsic asymmetry in brain dopaminergic systems interacts with left and right PFC lesions to differentially determine subcortical dopaminergic function and behavior that it subserves.

Whether our observation of an asymmetric rTMS-induced release of dopamine in specific medial prefrontal areas may be associated with the different modulatory effect on executive performance and behavior reported in previous TMS reports cannot be established in the current study because our subjects were healthy controls, completely at rest and not engaging in any form of task. In addition, to rule out potential confounding factors to our findings, behavioral ratings before and after the left or right DLPFC rTMS were measured and statistical analysis did not reveal any significant effect in different behavioral aspect.

In the interpretation of our results, we should also keep in mind that methodological factors may have prevented to detected dopaminergic changes in other cortical areas. In fact, neurochemically, the medial prefrontal cortex (in particular the ACC) is an area with dense DA receptor concentration [38], [62]. Post-mortem human brain studies with [^125^I]epidepride have shown that concentration of dopamine receptor in different cortical areas is considerably lower than in the ACC [62] and pharmacological challenges (methylphenidate, amphetamine, alfentanil) during PET using high-affinity dopaminergic ligands have confirmed these observations revealing detectable changes in cortical dopamine mainly in cingulate cortex [34], [40], [41]. Thus, in light of these reports, we should not exclude the possibility that low concentration of dopamine receptor may have prevented to detect reliable and significant radioactive signal in other cortical areas such as the DLPFC.

It is well known that dopamine can be released via two main different mechanisms [63], [64]: (1) transient or phasic dopamine release caused by dopaminergic neuronal firing and (2) sustained tonic dopamine release regulated by glutamatergic inputs on dopaminergic terminals. Grace has suggested that phasic dopamine release consists of large amplitude, transient increase of synaptic dopamine which is rapidly removed from the synaptic cleft by re-uptake. In contrast, extrasynaptic tonic dopamine release occurs slowly with a prolonged onset, delayed peak and extended duration [63], [64]. The entire process occurs over periods of tens of minutes to hours. It is possible that, given the methodological approach used in our study, the [^11^C]FLB 457 PET technique is most sensitive to extra-synaptic tonic dopamine release. Given the important role of dopamine in placebo and the potential implication of rTMS use on treatment, it would be natural to ask whether our findings could be explained by an effect of placebo. We believe that this is not case for several reasons. First, while placebo does well apply to a patient population where the expectation of clinical benefit does in fact play a major role [65], this does not have the same impact on healthy normal subjects where there is not expectation of improvement or clinical benefit. In addition, our healthy subjects were naïve to TMS and its role as potential tool for treatment. Second, the release of dopamine observed in our study was quite focal (limited to few specific regions) and seen only following left but not right DLPFC stimulation. This would not be the case if placebo would play a role where a more diffuse effect [66] and no difference in laterality would be observed. Third, no differences were observed in our studies on behavioral measures between the left and right stimulation. For all these reasons, we consider quite unlikely a potential role of placebo.

To our knowledge, this is the first study to provide evidence of extrastriatal DA modulation following acute rTMS of the DLPFC with its effect limited to the medial part of prefrontal cortex including pregenual, subgenual ACC and medial orbitofrontal cortex. PET imaging studies with dopaminergic high-affinity ligands combined with rTMS are a promising method for exploring specific cortical neural networks and their functional connectivity. Studies of neurochemical functions with rTMS may be useful for exploring hemispheric differences and the effects of these differences on striatal and extrastriatal dopaminergic system. This may help to better delineate the neurobiological effects of rTMS for the treatment of different neurological and psychiatric diseases.

## Materials and Methods

### Subjects and design

Seven right-handed young (2 females: 5 males; age, 23.3±5.6 years) healthy subjects were enrolled in this study. Exclusion criteria included history of psychiatric and/or neurological disorder (particularly epilepsy), any previous exposure to stimulant drugs, pregnancy and migraine. To rule out structural lesions in the brain and to provide anatomical reference for the analysis, T1-weighted high resolution MRI image (GE Signa 1.5 T, T1-weighted images, 1 mm slice thickness) where obtained for each subject. Written informed consent was obtained in all cases before study enrollment. The study protocols were approved by the Ethical Committee of the Center for Addiction and Mental Health Research, University of Toronto. Each subject underwent two PET scans performed on separate days. On one day, the subject received right DLPFC stimulation; on the other day, the subject received left DLPFC. The two sessions were counterbalanced. Behavioral ratings were collected before and after the rTMS during each PET session.

### rTMS

**Figure 3:**
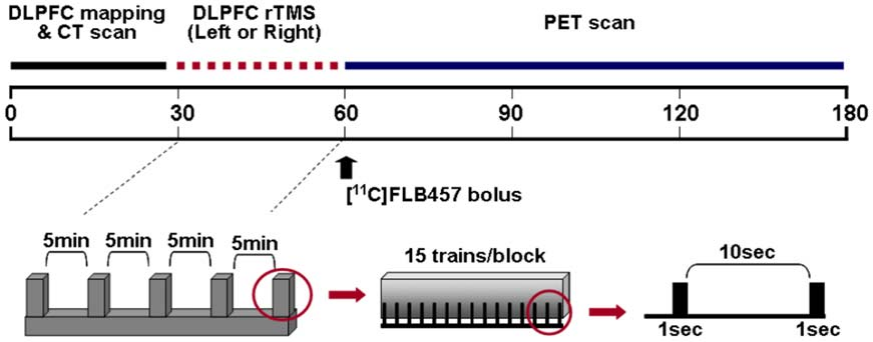
Timeline of the experimental setup for rTMS and [^11^C]FLB 457 PET imaging.

The rTMS procedure and target area (left/right DLPFC: [x, y, z] = [−/+40, 32, 30]) were similar to those used in our previous studies [11]. Biphasic high-frequency rTMS was carried out with the Magstim Rapid^2^ magnetic stimulator (Magstim, UK), using a figure-of-eight focal coil (70-mm diameter). The target area was localized on the individual MRI using the frameless stereotaxic system (Brainsight^TM^, Montreal, Canada). The TMS coil was held in the scanner in a fixed position by a mechanical arm over the target area and positioning was monitored under real-time guidance using the neuronavigation system. The coil was oriented so that the induced-electric current flowed in a posterior-anterior direction which has been considered to be more effective in activating underlying cortical neurons [67]. Five rTMS blocks were delivered, each block separated by a 5-minute interval. In each block, 15 trains of 1-sec duration were delivered at a stimulation frequency of 10 Hz, with a between-train interval of 10 sec. Thus, a total of 750 stimuli were delivered over about 30 minutes preceding the start of the PET acquisition (Figure 3). Stimulus intensity, expressed as a percentage of the maximum stimulator output, was set at 100% of the resting motor threshold for the contralateral first dorsal interosseous (FDI) muscle. Motor threshold was defined according to current guidelines [68], as the lowest stimulus intensity able to elicit 5 motor evoked potentials of at least 50 uV amplitude in a series of 10 stimuli delivered over the motor cortex at intervals longer than 5 seconds.

### PET Imaging

PET scans were obtained with a high resolution PET CT, Siemens-Biograph HiRez XVI (Siemens Molecular Imaging, Knoxville, TN, U.S.A.) operating in 3D mode with an in-plane resolution of approximately 4.6 mm full width at half-maximum. To minimize subject's head movements in the PET scanner, we used a custom-made thermoplastic facemask together with a head-fixation system (Tru-Scan Imaging, Annapolis). Following the acquisition of a scout view for accurate positioning of the subject, a low dose (0.2 mSv) CT scan for attenuation correction was conducted before the rTMS session. Within 5 minutes of the completion of the rTMS, 10mCi of [^11^C]FLB 457 were injected into the left antecubital vein over 60 seconds and 31 frames of dynamic scanning were acquired over 90 min [30]. For each 3D sinogram, data were normalized with attenuation and scatter corrected before applying fourier rebinning to convert the 3D sinograms into 2D sinograms. The 2D sinograms were then reconstructed into image space using a 2D filtered back projection algorithm, with a ramp filter at Nyquist cut-off frequency. After reconstruction a gaussian filter with a 5 mm FWHM was applied and the images calibrated to nCi/cc. The spatial resolution of the reconstructed images was 1.06×1.06×2 mm (X×Y×Z).

### Image analysis

**Figure 4:**
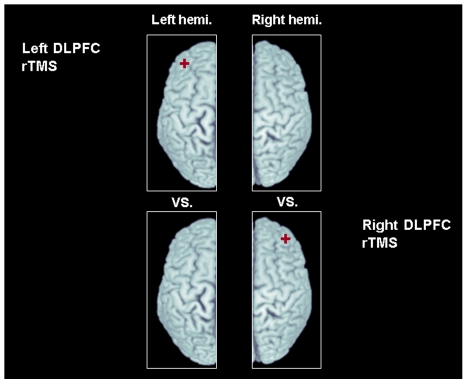
Contrast analysis for each stimulation conditions: changes in [11C]FLB 457 BP of the stimulated hemisphere were tested against [11C]FLB 457 BP of the ipsilateral (non-stimulated) hemisphere of the other session (control). Red mark indicate stimulation site.

After the realignment procedure for motion correction among the frames, motion corrected PET frames were summed, co-registered to the corresponding individual MRI and transformed into standardized stereotaxic space. The BP map in each subjects was generated by Pmod 2.9 (Pmod Technologies Ltd., Switzerland), based on the simplified reference model [69], [70] using the ipsilateral cerebellum of the stimulated hemisphere as a reference region. According to our previous studies [11], [12], [66], the effect of rTMS on dopamine release is strictly limited to the ipsilateral hemisphere thus changes in extrastriatal DA BP of the stimulated hemisphere were tested against [^11^C]FLB 457 BP of the ipsilateral (non-stimulated) hemisphere of the other session (control) (Figure 4). The image preprocessing and statistical analysis were done with SPM 2 (Wellcome Department of Imaging Neuroscience, London). Statistical maps were thresholded at a level of *P*<0.001 uncorrected with an extent threshold of at least 20 contiguous voxels [71]. Regions were considered significant at the threshold of *P*-value<0.05 corrected at the cluster level (z score>3.10). In the regions defined in our a priori hypothesis we performed a spherical volume correction (SVC radius 10 mm) and results were considered significant at cluster-based (family wise error - FWE) corrected P-value<0.05. A priori prefrontal areas were defined using the Brodmann's areas (BA 9/46, BA 24/25/32, BA 11) included in WFU_PickAtlas (SPM extension toolbox). To confirm individual changes in binding, BPs were extracted from regions of interest (ROI, radius = 10 mm) centered at the statistical peak defined by the parametric maps and a statistical analysis was performed with within-subject repeated-measures analysis of variance (ANOVA) with a 2 (rTMS site: left and right DLPFC rTMS)×2 (ROI site: left and right ROI) design for each of the three ROIs. A 2-tailed paired t-test (with Bonferroni corrections) was performed as post-hoc analysis to confirm the differences in BPs between rTMS and control condition for each ROI. Statistical analyses were performed with SPSS 13.0 software (SPSS Inc., Chicago, Illinois).

### Behavioral rating

Subjects completed behavioral questionnaires in which they rated the level of their comfort, fatigue, anxiety, mood, irritation and pain before and after each rTMS sessions. Ratings were made on a seven-point Likert scale ranging from −3 to 3, with −3 indicating the highest negative level and 3 indicating the highest positive level for each dimension.
